# Modulation of matrix metalloproteinase activity by EDTA prevents posterior capsular opacification

**Published:** 2012-06-22

**Authors:** Sarbani Hazra, Rajdeep Guha, Geram Jongkey, Himangshu Palui, Akhilesh Mishra, Geeta K. Vemuganti, Samar K. Basak, Tapan Kumar Mandal, Aditya Konar

**Affiliations:** 1 West Bengal University of Animal & Fishery Sciences, Kolkata, India; 2 CSIR- Indian Institute of Chemical Biology, Kolkata, India; 3 School of Medical Sciences, University of Hyderabad, Hyderabad, India; 4 Disha Eye Hospitals & Research Centre, Barrackpore, Kolkata, India

## Abstract

**Purpose:**

To evaluate the effect of ethylenediaminetetraacetic acid (EDTA) on posterior capsular opacification (PCO) of rabbits and to assess its effect on intraocular tissues.

**Methods:**

Modulation of matrix metalloproteinase (MMP) activity in the aqueous following cataract surgery in rabbits and its prevention by different doses of EDTA was determined by zymography. For evaluation of PCO, lensectomized rabbits were intracamerally injected with single dose of either 5 mg EDTA or normal saline. After one month, the degree of PCO was determined by slitlamp biomicroscopy, Miyake-Apple view, and histology of the lens capsule. The effect of EDTA on intra ocular pressure (IOP), corneal endothelial cells, and the retina was evaluated by tonometry, specular microscopy and scanning electron microscopy, and electroretinography. The concentration of EDTA in the aqueous was determined by high performance liquid chromatography (HPLC) at different time points.

**Results:**

The MMP activity was significantly increased in the aqueous of the operated eyes, and EDTA reduced the degree of increase in a dose-dependent manner. EDTA treatment significantly reduced the degree of PCO (p<0.05). Histopathology of the lens capsule showed a reduction in the number of proliferating and migrating cells as well as MMP2 expression in the EDTA-treated eyes. EDTA treatment did not change the IOP; density, morphology and ultrastructure of the corneal endothelial cells; and electroretinography (ERG). EDTA was detectable in the aqueous humor up to 72 h following a single intracameral injection.

**Conclusions:**

EDTA reduces the degree of PCO by suppressing the MMP activity and it is not toxic to intra ocular structures at the concentration used.

## Introduction

Posterior capsular opacification (PCO) remains a major complication of extracapsular cataract surgery and can lead to severe vision impairment. The improvements in the surgical procedures and alterations in the design of the implanted intraocular lenses have led to a reduction in the rate of PCO [1]. However, the rates still remain high [2].

The current treatment for PCO is YAG laser capsulotomy but this procedure can lead to several complications i.e., intraocular lens (IOL) optic damage/pitting, postoperative intraocular pressure (IOP) elevation, cystoid macular edema, retinal detachment, and IOL subluxation [1]. Therefore, there is a strong need to determine how to prevent PCO [1]. The major thrust of PCO prevention experiments has been toward the use of pharmacological agents that block the formation of PCO. A major drawback of this approach has been the toxicity of the drug on the healthy tissues of the eye [3].

PCO is believed to develop when the lens epithelial cells (LECs) remaining in the capsular bag after cataract surgery undergo epithelial-mesenchymal transition stimulated by the inflammatory cytokines and growth factors that are expressed after cataract surgery. This transition results in their ability to move and migrate to the posterior capsule and cause wrinkling, matrix production, and contraction. This then leads to opacification of the capsule and significant vision reduction [4].

The matrix metalloproteinases (MMPs) are a multigene family of tightly regulated zinc-dependent enzymes. They are key modulators of important biologic processes that are expressed during physiologic events, e.g., skeletal formation, angiogenesis, cellular migration, inflammation, wound healing, and cancerous growth [5]. They are found in normal tissues and are overexpressed under various pathological conditions including excessive scarring [6]. The MMPs play a major role in the cleavage of the protein components of the extracellular matrix by the proteolysis of the matrix which leads to cell migration through the matrix. Matrix modification and transdifferentiation of LECs have been observed following MMP induction by transforming growth factor beta 2 (TGF-β2) in cultured cells and in human lens capsular bags [7-9]. An upregulation of MMPs has been observed after experimental cataract surgery [10], and a down-regulation of MMP-2 and MMP-9 has been detected by proteasome inhibition. This inhibition led to a blockage in the migration of LECs and prevention of PCO [1]. Inhibition of the MMPs by ilomastat (GM6001), a MMP inhibitor, also prevented the migration of human LECs and contraction of the lens capsule in isolated human lens capsule in vitro [11]. Thus, MMP inhibition is a potential target for PCO prevention.

Ethylenediaminetetraacetic acid (EDTA) is a strong chelator of metal ions and a broad spectrum MMP inhibitor. Its effect on PCO prevention has not been fully explored, although preliminary results have suggested that EDTA can block LEC migration to the posterior capsule [12,13]. There is need for a comprehensive study on the effects of inhibiting MMP in the aqueous humor by EDTA on PCO.

Thus, the purpose of this study was to determine the effect of a single intraocular injection of EDTA immediately after cataract surgery on PCO formation. To accomplish this, we performed lensectomy by phacoemulsification in rabbits, and treated one-half of the eyes with EDTA and the other half with saline. After a fixed time, we compared the degree of PCO in the two groups. We also determined the post injection concentration of EDTA in the aqueous, and its effect on the intraocular pressure, density, morphology, and ultra structure of the corneal endothelial cells, and physiology of the retina.

## Methods

This study was performed in accordance to the guidelines of the Committee for the Purpose of Control and Supervision of Experiments on Animals (CPCSEA) of the Government of India. This study was specifically approved by the “Institutional Animal Ethics Committee” (Approval No.EC268) registered with CPCSEA (Registration no 763/03/a/CPCSEA,dated 5.6.2003), Department of Pharmacology & Toxicology, Faculty of Veterinary & Animal Sciences, West Bengal University of Animal and Fishery Sciences, Kolkata, India. The procedures used conformed to the guidelines of Association of Research in Vision and Ophthalmology on animal usage.

### Animals

**Figure 1:**
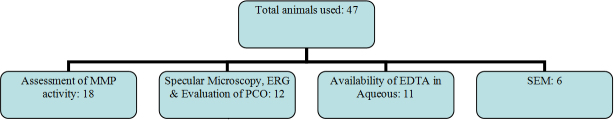
Animal usage for the study. Flow chart shows number of animals used for different experiments.

The experiments were conducted on 47 healthy, adult New Zealand White rabbits of about one–year-of-age and weighing around 2 kgs. The distribution of animal usage in different experiments is presented in the flowchart (Figure 1).

### Surgical methods

This was an unmasked, non-randomized animal experimental study. All surgical procedures were performed by the same surgeon (S.H.). The animals were anesthetized with 6 mg/kg of xylazine hydrochloride (Xylaxine^®^; Indian Immunologicals Ltd., Hyderabad, India) and 30 mg/kg of ketamine hydrochloride (Ketamine 50^®^; Themis, Haridwar, Uttarakhand, India). A retrobulbar injection of 4% lignocaine hydrochloride was made into the extraconal space for local anesthesia and also to prevent eye movements. The pupil was dilated with topical tropicamide eye drops (Tropicacyl^®^, 1% w/v; Sunways (India) Pvt. Ltd., Mumbai, Maharastra, India) and phenyephrine eye drops (Ocurest^®^ 0.12%; Centaur Pharmaceuticals Pvt. Ltd., Mumbai, India). The ocular surface was disinfected with 5% povidone-iodine, and the lids were retracted with a lid speculum.

A clear corneal incision was made with a 2.8 mm keratome, and 0.1 ml air was injected to prevent staining the corneal endothelium. The anterior capsule was stained with 0.08% trypan blue, and a visco elastic solution of 2% hydroxypropyl methyl cellulose (Appavisc® 2% w/v; Appasamy ocular devices, Pvt. Ltd. (Pharma division),Vadamangalam, Puducherry, India) was used to reconstitute the anterior chamber. A continuous curvilinear capsulorrhexis of approximately 5.5 mm was performed with a capsular forceps.

The phacoemulsification tip was inserted through the corneal incision, and the nucleus was removed by the “chip-and-flip” technique with an ultrasound power of 60% to 70%, an aspiration flow rate of 16 to 20 ml/min, and vacuum of 80 to 120 mmHg. All cortical materials were carefully and completely removed by irrigation/aspiration.

Before the placement of the last corneal suture, either 0.5 ml solution containing different concentrations of EDTA or 0.5 ml of normal saline was injected into the capsular bag. The postoperative treatment consisted of topical flubiprofen, ciprofloxacin, and prednisolone eye drops.

### Assessment of MMP activity

Lensectomy by phacoemulsification was performed on one eye of New Zealand White rabbits, and at the end of surgery 0.5 ml solution containing either 1 mg of EDTA or equal volume of normal saline was injected into the capsular bag of six animals. After 24 h, 0.2 ml of aqueous from EDTA treated, normal saline treated and unoperated fellow eyes were drawn from the anterior chamber with a 26 gauge needle and collected in centrifuge tubes separately. MMP activity in the aqueous was measured by zymography and compared. In another two sets of similar experiments using 6 rabbits in each set, MMP activity was measured using 2.5 mg and 5 mg EDTA and compared with corresponding saline treated controls. In each set of experiment aqueous from the fellow eyes were used to determine the normal MMP activity in the aqueous of unoperated eyes. Topical 0.5% proparacaine hydrochloride was used as a local anesthetic before each aqueous collection.

### Zymography

Zymography was performed following a standard protocol. In brief, 10% SDS-polyacrylamide gel was prepared by copolymerizing with 0.1% bovine gelatin (Sigma, St Louis, MO). Then, 3 µl of aqueous from the individual eyes were mixed with equal volume of 2× sample buffer, loaded onto the gel without boiling and electrophoresed under non- reducing condition at 4 °C. The gel was placed in 2.5% Triton X (Sigma) at room temperature on a shaker to remove the SDS and incubated overnight at 37 °C in a reaction buffer (50 mM Tris, pH 7.5; 10 mM Cacl_2_) to allow proteinase digestion of the substrate. The gel was then stained with Coomassie brilliant blue (Sigma), which showed the positions of the enzyme activity as clear bands on a blue background. The band intensities were quantified by imageJ software (NIH). The results are expressed as fold change compared to MMP activity in the normal aqueous.

### Evaluation of PCO formation

Assessment of the degree of PCO was done with 5 mg dose of EDTA as we observed maximum MMP inhibition with this dose. Lensectomy by phacoemulsification was done on one eye of 12 New Zealand White rabbits and each eye was injected with either 0.5 ml solution containing 5 mg EDTA or normal saline. Eyes were examined regularly by slit-lamp biomicroscopy (Topcon, Tokyo, Japan). After one month, the degree of PCO in the EDTA-treated and saline-treated control groups was graded on a scale of 0 to 3: 0=clear, no visible proliferative tissue on the peripheral or central capsule; 1=mild, proliferative tissue only in the periphery; 2=moderate, sparse proliferative tissue on the peripheral and central capsules; and 3=severe, dense, diffuse, and thick opacification on the entire capsule [13].

### Measurement of intraocular pressure (IOP)

The intraocular pressure (IOP) was recorded before surgery and at 6 h, 2 days, and 7 days postoperatively by applanation tonometer (TONO-PEN®XL, Reichert, Depew, NY). Three recordings were taken from each eye, and the average was used for the statistical analyses.

### Specular microscopy

Two weeks following surgery, corneal endothelial cell density and cellular morphology of the control and EDTA treated eyes was determined by specular microscopy.

### Electroretinography (ERG)

Electroretinography was performed on EDTA-treated eyes two weeks after surgery and the ERG values obtained from the same eyes before surgery were considered as baseline values. The rabbits were dark-adapted for 2 h, and then one drop of 1% tropicamide and 2.5% phenylephrine was applied topically on both eyes for pupillary dilatation. They were anesthetized with the same anesthetics as described previously. One drop of methylcellulose (Appavisc®2% w/v; Appasamy ocular devices, Pvt. Ltd. (Pharma division),Vadamangalam, Puducherry, India) was applied topically to the eye. ERG recordings were conducted as recommended by the ISCEV guidelines (Reti Animal, Roland Consultancy, Friedrich-Franz, Brandenburg-an der Havel, Germany). The body temperature of the animals was maintained at 37 °C throughout the experiment. The electrodes were placed under dim red light. The active gold ring electrode was placed on the cornea, and the reference electrode (sub dermal needle electrode) was placed subcutaneously between the base of the ear and lateral canthus. The ground electrode was placed subcutaneously just on the occiput (on top of the head).

### Gross histology

After one month, the rabbits were euthanasized by an overdose of barbiturate. The eyes were enucleated, and placed in 10% formalin for 48 h. The eyes were sectioned in the coronal plane just anterior to the equator. Miyake-Apple view photographs of the posterior capsule were taken. The entire capsule was removed for histopathology.

### Histopathology

The lens capsules were fixed in 10% formalin, embedded in paraffin, and cut into 5 µm-thick sections. Sections were stained with hematoxylin-eosin and examined under light microscope (Leica DM 2500; Leica Microsystems GmbH, Wetzlar, Germany) for the evaluation of cellular proliferation and migration to the posterior capsule. Slides were photographed under 20× magnifications and number of cells/100 µm of lens capsule was counted.

### Immunohistochemistry

Paraffin sections were deparaffinized, rehydrated, kept at room temperature in 10 mM sodium citrate for 10 min, and then antigen retrieval was performed by pressure-cooking for 2 min in 0.01 m sodium citrate, pH 6 [14]. Nonspecific binding was blocked by placing the sections in 6% BSA for 4 h in a humidified chamber. Samples were washed three times in PBST containing 2% BSA for 5 min, incubated overnight in a humidified chamber with MMP-2 primary antibody (1:50, Sc-10736; Santa Cruz Biotechnology, Santa Cruz, CA), and washed three times with PBST containing 2% BSA for 5 min. The slides were incubated in a humidified chamber with an anti rabbit secondary antibody tagged with Alexa Fluor (1:100, A10042; Invitrogen, Carlsbad, CA) for 2 h, and washed three times with PBST containing 2% BSA for 5 min. The sections were stained with DAPI (4'-6-diamidino-2-phenylindole). The immune complexes were examined under a fluorescence microscope (Leica DM 2500; Leica) and photographed under the same (20×) magnification. The fluorescence intensity was quantified by image J software.

### Scanning electron microscopy of the cornea

Scanning electron microscopy of cornea was performed on 6 other animals following lensectomy and injection of either 5 mg EDTA or normal saline as described earlier. Cornea from both EDTA treated (n=3) and control eyes (n=3) were preserved in 2.5% glutaraldehyde. Following dehydration in graded alcohol, all specimens were critical point dried, coated with gold (IB-2 ion coater; Eiko Engineering, Ibaraki, Japan), and observed under scanning electron microscope (SEM; Hitachi S530; Hitachi, Tokyo, Japan).

### Determination of EDTA in aqueous humor

Five mg of EDTA was injected in 21 eyes immediately after lensectomy and at different time points, 0.2 ml aqueous was collected in separate vials containing 0.8 ml of acetonitrile (ACN) solution for detection of EDTA in the aqueous by HPLC. Topical 0.5% proparacaine hydrochloride was used as a local anesthetic before each aqueous collection.

### Statistical analyses

The significance of differences between two groups was determined by one way anova, and the significance level was set at p<0.05.

## Results

All animals survived the study period of one month, and no major postoperative complications were encountered. The inflammation post operatively in control as a well as in treated animals was not remarkable, there was absence of post operative blepharospasm or signs of discomfort from one day after surgery in all the animals. We observed mild corneal edema postoperatively in both the control and treated eyes which resolved within 2–3 days.

### EDTA suppressed MMP2 activity

**Figure 2:**
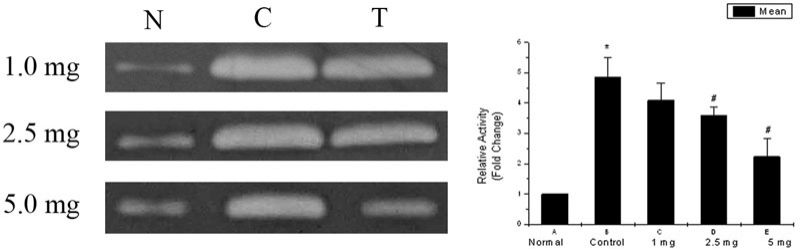
Effect of EDTA on the MMP2 activity in the aqueous humor of rabbit eyes. Gelatine zymography of the aqueous from un-operated normal (N), saline injected control (C), and EDTA-treated (T) eyes shows an increase in the MMP2 activity in control eyes and the increase is reduced by EDTA. *p<0.05 versus Normal; ^#^p<0.05 versus Control. A&B-n=9; C,D&E-n=3.

Gelatine zymography (Figure 2; Table 1) showed significantly higher MMP2 activity in the aqueous humor of the eyes that underwent surgery and injection of saline than in the fellow unoperated eyes. The injection of 1 mg EDTA did not alter the increased MMP2 in the operated eyes. However, the MMP2 activity was significantly reduced following injection with 2.5 mg and 5 mg of EDTA.

**Table 1:** MMP2 activity determined by gelatine zymography.

**MMP2 activity**	**Normal**	**Control**	**1 mg EDTA**	**2.5 mg EDTA**	**5 mg EDTA**
(Relative activity/fold change)	1	4.8±0.20*	4.1±0.32	3.6±0.15#	2.2±0.36#

Gelatine zymography shows that the MMP2 activity in the aqueous of operated (control) animals increased compared to un-operated (normal) animals and the increased MMP activity was reduced following EDTA treatment. *p<0.05 versus Normal; ^#^p<0.05 versus Control. A and B-n=9; C, D, and E-n=3.

### EDTA did not influence the IOP

The mean±SD IOP recorded by applanation tonometer (TONO-PEN^®^XL; Reichert) at the baseline were 10±1.58 mmHg and 9.67±1.80 mmHg in the treatment and control group, respectively. The IOPs of the EDTA treated-eyes was 9.96±0.86 and that in the saline-treated eyes was 9.97±0.45 mmHg at 6 h. At 2 days, the mean IOP was 9.8±1.40 in the EDTA-treated eyes and 9.0±0.96 mmHg in the saline-treated eyes. At 7 days, the mean IOP was 9.75±0.78 mmHg in the EDTA-treated eyes and 8.9±1.33 mmHg in the saline-treated eyes. None of these differences was significant (p>0.05).

### Slit lamp biomicroscopy and Miyake Apple view showed reduction in PCO formation following treatment with EDTA

**Figure 3:**
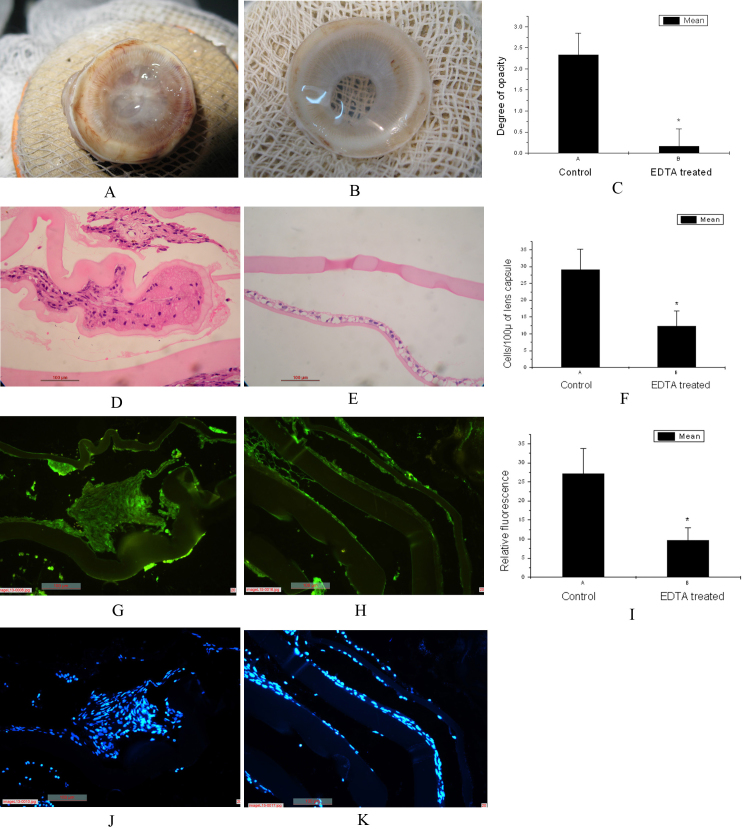
Effect of EDTA on posterior capsular opacification. Miyake Apple posterior view shows opacification of posterior capsule in control eyes (**A**), a clear posterior capsule in EDTA treated eyes (**B**); and quantitative representation for degree of opacification (**C**). Histological section of the lens capsule from control (**D**) eyes shows increased number of LECs arranged in multilayers whereas reduced cellular proliferation in EDTA-treated eyes (**E**); and quantitative representation (**F**). Immunohistochemistry shows increased expression of MMP2 in the lens capsule from control eyes (**G**) and reduced expression in the lens capsule from EDTA treated eyes (**H**), quantitative representation (**I**), corresponding DAPI stained sections (**J**) and (**K**). Magnification: 20×; p<0.05.

One month postoperatively, a significant (p<0.05) reduction in the degree of PCO was observed in the EDTA-treated group than in the saline-treated control group under slit-lamp biomicroscope. The PCO scores of the EDTA-treated eyes was 0 in 5 eyes and 1 in 1 eye, whereas the comparable scores was 2 in 4 eyes and 3 in 2 eyes (Table 2; p<0.05). A qualitative evaluation of the lens capsule through a posterior view using the Miyake-Apple posterior photographic technique also showed a clearer posterior capsule in the treatment group (Figure 3B) and an opaque posterior capsule in the control group (Figure 3A).Thus, both slit-lamp biomicroscopy and Miyake-Apple posterior view showed a significant reduction in the degree of PCO in the eyes treated with EDTA.

**Table 2:** PCO score Control versus EDTA.

**PCO**	**Control**	**EDTA**
0, clear	-	5
1, mild	-	1
2, moderate	4	-
3, severe	2	-
PCO (Mean±SD)	2.33±0.5	0.167±0.4

Phacoemulsification was done in one eye of 12 New Zealand White rabbits and injected with either 0.5 ml solution containing 5 mg EDTA or normal saline. After one month PCO formation was evaluated by slit lamp microscopy and scored. Data are expressed as Mean±SD, n=6, p<0.05.

### Histology of lens capsules indicated PCO prevention with EDTA treatment

Histological sections of the lens capsules from control eyes (Figure 3D) showed a large number of LECs in the anterior capsule, and the cells were arranged in multilayers. Migration of LECs and their deposition on the acellular posterior capsule was also observed. But a single layer of epithelial cells was observed in the anterior capsule and acellular posterior capsule in the histological sections of lens capsules from EDTA-treated eyes (Figure 3E).These findings indicated that EDTA prevented cellular proliferation and migration.

### Expression of MMP2 was reduced in EDTA treated lens capsules

Immunostaining showed a weak expression of MMP2 in the sections of lens capsule from the EDTA-treated group (Figure 3H), but a significantly stronger expression in the saline-treated group (Figure 3G).We found that an increase in the proliferation and migration of the LECs was accompanied by increased expression of MMP2. Similarly, less proliferation and migration of LECs was observed in EDTA-treated eyes with weak expression of MMP2.

### Specular microscopy indicated no adverse influence of EDTA on corneal endothelium

**Figure 4:**
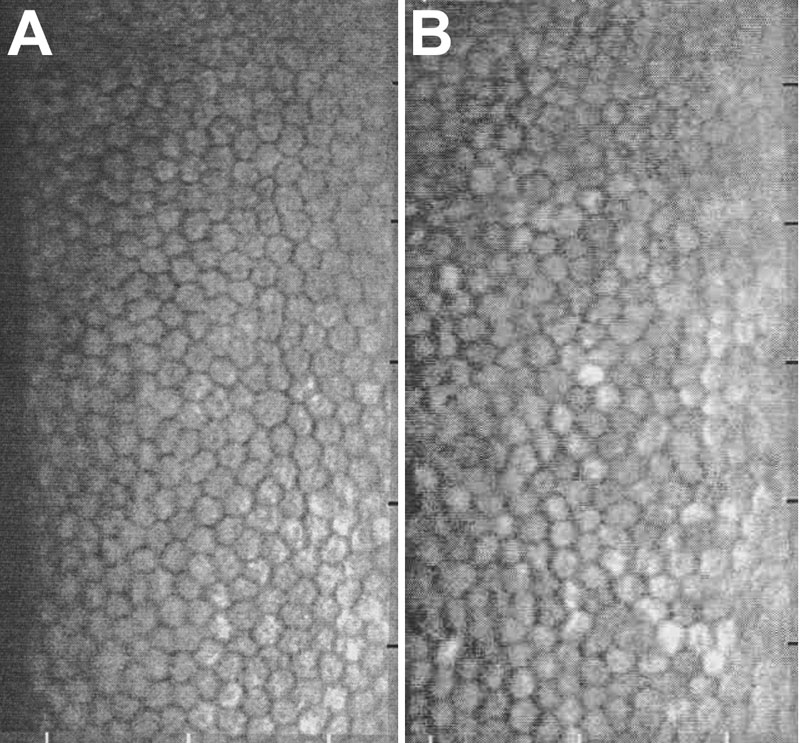
Effect of EDTA on the corneal endothelial cells. Specular microscopy shows no decrease in the density of corneal endothelial cells and no change in cellular morphology in both control (**A**) and EDTA treated (**B**) eyes.

Specular microscopy showed no significant decrease in the corneal endothelial cell density in the EDTA-treated eyes. The mean±SEM cell density in the control eyes was 2483.6±152.8 cells/mm^2^ which was not significantly different from the 2213.5±114.4 cells/mm^2^ in the EDTA-treated eyes (p>0.05). No change in the morphology of the cells was observed in the EDTA-treated eyes (Figure 4B) compared to the control eyes (Figure 4A).

### EDTA treatment did not change the ultra structure of corneal endothelial cells

**Figure 5:**
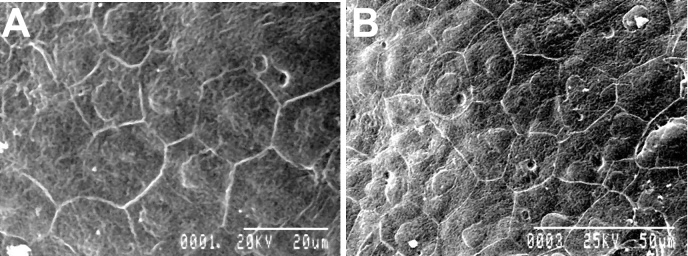
Scanning electron microscopy shows well defined corneal endothelial cells with unaltered cellular junction in both control (**A**) and EDTA treated (**B**) eyes.

Scanning electron microscopy of cornea from both control (Figure 5A) and EDTA treated (Figure 5B) eyes showed continuous layer of polygonal endothelial cells with well defined cellular junction. This confirms that there was neither any cellular loss nor any change in the cellular architecture and integrity of the cellular junction due to EDTA treatment.

### Electroretinography values were not affected following treatment with EDTA

**Figure 6:**
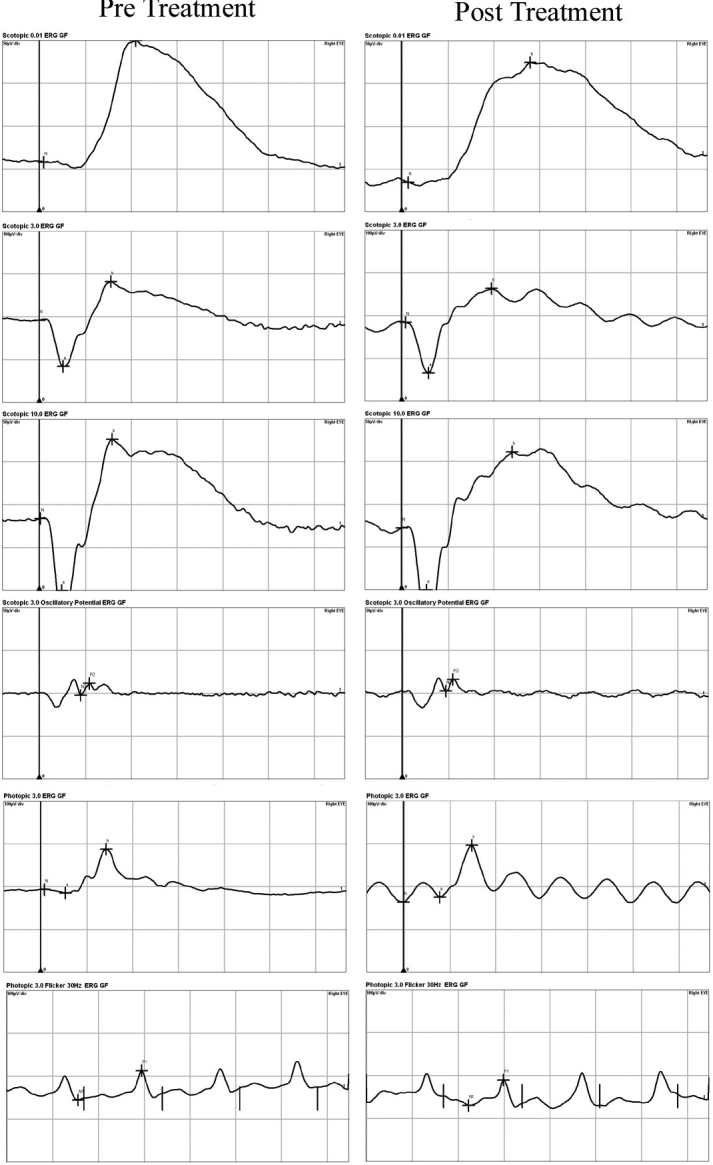
Effect of EDTA on the electroretinogram. No significant change in the b-wave amplitudes and implicit times can be seen after 5 mg of EDTA treatment compared to baseline values (n=6).

The ERGs recorded two weeks following drug treatment were compared to those at the baseline to determine if any significant changes had occurred in the retinal physiology. The means ± SEMs of the scotopic b-wave amplitudes and implicit times elicited by different stimulus intensities and recorded before and after EDTA treatment (Table 3) are plotted in Figure 6. None of the differences was significant (p>0.05; n=6; Table 3). Similarly, the differences in the amplitudes and implicit times of the photopic b-waves were not significant. The photopic flicker ERGs were not significantly different (p>0.05; n=6).

**Table 3:** ERG values recorded before surgery and 2 weeks post operatively from EDTA-treated eyes.

** **	**b-wave amplitude**		**b-wave implicit time**	
**Stimulus intensities**	**Before operation and EDTA treatment (base line values)**	**After operation and EDTA treatment**	**Before operation and EDTA treatment (base line values)**	**After operation and EDTA treatment**
Scotopic 0.01	101.85±11.8	148.3±25.6	54.8±5.4	55.3±2.5
Scotopic 3.0	182.5±24.9	223.2±30.6	36.8±2.1	37.8±1.4
Scotopic 10.0	193.0±27.8	236.0±32.6	36.6±1.4	42.3±2.8
Photopic 3.0	92.8±12.9	114.3±9.8	29.1±0.6	29.0±0.4
** **	N1		P1	
Photopic 30Hz flicker	Before EDTA treatment (base line values)	After EDTA treatment	Before EDTA treatment (base line values)	After EDTA treatment
** **	37.6±2.5	41.6±2.1	60.5±0.76	60.2±0.16

The ERG values recorded 2 weeks after operation and EDTA treatment did not vary significantly (p>0.05) from the base line values, recorded before operation and EDTA treatment. Data are expressed as Mean±SEM; n=6.

### Presence of EDTA in aqueous

**Figure 7:**
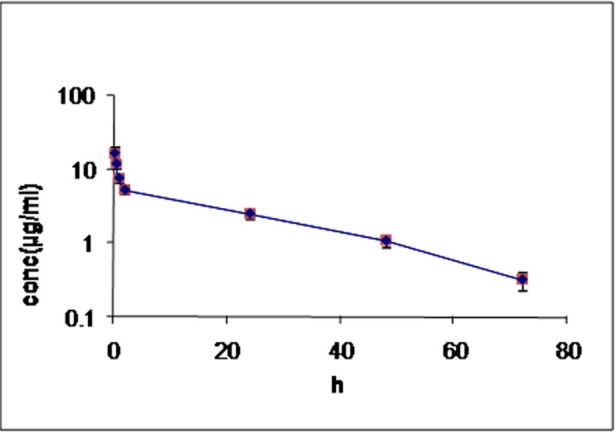
Concentation of EDTA in the aqueous humor after an intraocular injection of 5 mg of EDTA. The concentration of EDTA in the aqueous humor is plotted on the ordinate and time on the abscissa. Data are the means±SEM (n=3).

The mean aqueous concentration of EDTA is plotted as a function of time (Figure 7). EDTA was available in the aqueous humor for up to 72 h following single intracameral injection of 5 mg.

## Discussion

Our results showed an increase in the MMP activity in the aqueous following cataract operation, and the increase was reduced by a single intracameral injection of EDTA in rabbits.The suppression of MMP activity resulted in a decrease in the migration of LECs to the posterior capsule that prevented the development of PCO. We also observed that EDTA did not cause any change in the IOPs and density and morphology of the corneal endothelial cells. The retinal electrophysiology was normal following EDTA treatment and EDTA remained available in the aqueous for up to 72 h following a single intraocular injection.

There are several studies that showed an increase of the MMP activity after cataract surgery, and the authors suggested that MMP inhibition might be a good way to prevent PCO formation [1,10,11]. GM6001, a chelator of metal ions, is reported to prevent the migration and contraction of human epithelial cells of the lens capsule by inhibiting MMP in vitro [11]. Like GM6001, EDTA also inhibits MMP activity through chelation of metal ions. The degree of inhibition increases with the number of chelate rings. EDTA is hexadentate (“six-toothed”) ligand and forms six chelate rings compared to GM6001, which is bidentate. We tested EDTA because of its predicted stronger suppression of MMP activity. Preliminary reports suggested that EDTA prevented LEC migration to the posterior capsule [12,13].

Our findings show that lensectomy by phacoemulsification in rabbits can be a good model to study PCO and can be used to test different therapeutics. Rabbits are also a good choice because PCO develops fast in rabbits [15]. We have used rabbits of approximately the same age for the entire study because differences in age can be a confounding factor in the formation of PCO.

Our zymography data showed that there was a significant increase in the MMP2 activity in the aqueous of saline-treated control animals after cataract surgery. This increase was reduced by intraocular EDTA in a dose-dependent manner.

EDTA, doxycyclin, and NAC (n-acetylycysteine) have been shown to inhibit MMPs by chelating zinc which is required as a cofactor with calcium as stabilizers of the MMPs [16]. EDTA causes chelation of the calcium ion and interferes with the stability of the MMPs and has been reported to have high MMP inhibition activity in vitro [17]. Topical EDTA has been used to treat corneal ulcers by inhibiting MMPs, and promising results have been reported [18].

The volume of the drug injection was selected to be 0.5 ml because the combined volume of the anterior chamber (0.30 ml), posterior chamber (0.06 ml), and lens (0.20 ml) [19] following lens extraction including the empty capsular bag is about 0.56 ml.

We found a significant decrease in the degree of PCO in EDTA-treated eyes under slit-lamp microscopy which was consistent with the findings of gross histology (Miyake Apple posterior view). The number of proliferating and migrating LECs was reduced in the EDTA-treated eyes. Histological sections of lens capsule from control eyes showed an increased number of LECs in the anterior capsule arranged in multilayer and the presence of LECs in the posterior capsule. The single layer of LECs in the anterior capsule and acellular posterior capsule indicated a marked decrease of PCO in the EDTA-treated animals.

The postoperative increase in MMP activity resulted in a degradation of matrix and increased migration of LECs to the posterior capsule. These changes were abrogated by EDTA by its ability to reduce MMP activity. Nishi et al. [12] and Inan et al. [13] also observed decreased cellular migration to the posterior capsule after EDTA treatment and predicated an inhibition of the degradation of cadherin junctions might be the reason for this. We have observed reduced expression of MMP2 in histological section of the lens capsule from EDTA-treated eyes compared to the control eyes. This can be due to fewer migrating cells in the posterior capsule of EDTA-treated eyes which resulted in less secretion and weaker expression of MMP2 by these cells than the cells in the control eyes.

Toxicity of the intraocular tissues remains a major concern when drugs are injected into the eye for PCO prevention. Various cytotoxic and anti-metabolite drugs such as methotrexate, mitomycin C, daunomycin, 5-flurouracil, colchicines, and daunorubicin have proved to be effective in inhibiting proliferation of LECs in vitro, but none of them has been used clinically due to their possible toxic effects [3,13]. Although earlier studies reported corneal edema following EDTA treatment [13], we observed only mild corneal edema that resolved within 2–3 days, and the opacities were present in both the EDTA-treated and saline-treated groups [20]. Thus, we can conclude that the corneal edema was surgery induced. We conducted a separate set of experiments where EDTA was injected without performing phacoemulsification and corneal edema was not observed. Thus, the edema must have been induced by the surgical procedures.

MMP is involved in the turnover of matrix and is required for maintenance of the IOP. Down-regulation of MMP activity has been thought to be the mechanism for the pathophysiology of open angle glaucoma [21-23]. However, we did not observe any change in the IOP following EDTA treatment. This may be because the EDTA treatment did not decrease the MMP activity below normal level. We did not observe any change in the density or morphology of the corneal endothelial cells which indicated that EDTA was not toxic at the dose used. Further, scanning electron microscopy of the cornea also confirmed that EDTA treatment did not change the architecture of the endothelial cells and integrity of the intercellular tight junction. The amplitudes and implicit times of the b-waves of the scotopic and photopic ERGs after EDTA treatment did not differ significantly from their baseline values. Thus, EDTA did not induce any toxic effect to retina. A limitation of this study was that the evaluators were unmasked.

The maximum non-toxic dose of EDTA to endothelium and retina needs to be determined before preclinical further studies. Intraocular lens (IOL) was not implanted in the present study; therefore, effect of EDTA on various biomaterials of IOL also needs evaluation.

We conclude that EDTA suppresses the post surgical enhanced MMP activity in the aqueous and thereby prevent posterior capsular opacification. It does not show any adverse effect to the intraocular structures. Therefore, it might be an option for therapeutic prevention of PCO.
